# Titanium Immobilized with an Antimicrobial Peptide Derived from Histatin Accelerates the Differentiation of Osteoblastic Cell Line, MC3T3-E1

**DOI:** 10.3390/ijms11041458

**Published:** 2010-04-02

**Authors:** Seicho Makihira, Takahiro Shuto, Hiroki Nikawa, Keishi Okamoto, Yuichi Mine, Yuko Takamoto, Masaru Ohara, Koichiro Tsuji

**Affiliations:** 1 Division of Oral Health Sciences, Graduate School of Biomedical Sciences, Hiroshima University, 1-2-3 Kasumi, Minami-ku, Hiroshima 734-8553, Japan; E-Mails: shuto-tin@hiroshima-u.ac.jp (T.S.); hirocky@hiroshima-u.ac.jp (H.N.); yuichimine@hiroshima-u.ac.jp (Y.M.); b065773@hiroshima-u.ac.jp (Y.T.); 2 Toyo Advanced Technologies Co., LTD. 5-3-38, Ujina-Higashi, Minami-ku, Hiroshima 734-8501, Japan; E-Mail: okamoto.ke@toyo-at.co.jp (K.O.); 3 Hiroshima University Hospital, 1-2-3 Kasumi, Minami-ku, Hiroshima 734-8553, Japan; E-Mail: mohara@hiroshima-u.ac.jp (M.O.); 4 Two cell Co. LTD., 1-2-3 Kasumi, Minami-ku, Hiroshima 734-8553, Japan; E-Mail: ktsuji@hiroshima-u.ac.jp (K.T.)

**Keywords:** antimicrobial peptide, titanium surface, MC3T3-E1 cells

## Abstract

The objective of this study was to evaluate the effect of titanium immobilized with a cationic antimicrobial peptide (JH8194) derived from histatin on the biofilm formation of *Porphyromonas gingivalis* and differentiation of osteoblastic cells (MC3T3-E1). The titanium specimens (Ti) were immobilized with JH8194, according to the method previously described. The colonization of *P. gingivalis* on JH8194-Ti was significantly lower than that on control- and blocking-Ti. JH8194-Ti enhanced the mRNA expressions of Runx2 and OPN, and ALPase activity in the MC3T3-E1, as compared with those of control- and blocking-Ti. These results, taken together, suggested the possibility that JH8194-Ti may be a potential aid to shorten the period of acquiring osseointegration.

## 1. Introduction

Dental implant treatment has been developed and established all over the world, since the principles were proposed by a Swedish group and it has been reported that the rate of success of dental implant treatments in controlled patients is very high, over 90% [1,2]. However, reports on failures of implants resulting from excess load and periimplantitis, have been increasing in number [3,4]. Esposito *et al*. reviewed the causes of early implant failures, based on the histological, clinical and radiographic findings. It was concluded that three major etiologies may be implicated in the failure process: impaired healing ability of the host bone site, disruption of a week bone-to-implant interface after abutment connection, and infection with complicated surgery [5,6]. These suggested that requirements for increased success of dental implants might be the development of materials to enhance osseointegration and to protect against early bacterial infection, as well as the establishment of easier surgical procedures.

Titanium is the main material for dental implants, since titanium possesses excellent physical properties, and is suitable for acquiring osseointegration easily. The titanium surface is formed with a dioxide film, which increases calcium deposition and is readily reactive towards an osseous protein. Therefore, some changes occur to the titanium surface properties, and technological manipulations to achieve a shortening of the period of osseointegration acquisition and maintenance of strong osseointegration are widely done [7]. Taking advantage of this characteristic of the titanium surface, we previously attempted its modification. Concretely, in order to control osteoclast differentiation around dental implants, we produced titanium specimens immobilized with osteoprotegerin (OPG), which was a decoy receptor for one of the osteoclast-inducing factors; receptor activator of NF-κB ligand (RANKL), using our immobilization-method [8]. This titanium blocked osteoclast differentiation, indicating the function of OPG remained after the immobilization process, which indicated that the immobilization strategy should be available for other proteins and synthesized peptides [8].

Recently, we have demonstrated that the cationic synthetic peptide; JH8194, that is a histatin analog, has powerful anti-candicidal activity [9]. Furthermore, it was reported that histatin 5 synergistically increased the proliferation of chondrocytes under the epidermal growth factor [10]. The peptides derived from histatin and lactoferricin exerted cytotoxic effects on MC3T3 at high concentration (400 μg/mL) [11]. These reports suggested that antimicrobial peptides or synthetic peptides derived form histain or lactoferricin would have biological function on mammalian cells, including osteoblast and chondrocyte cells. Additionally, the inhibitory activity of histatin against hemaagglutinating activities of *P. gingivalis*, which was the pathogenic bacteria isolated from periodontitis and periimplantitis, was shown [12]. Based on these findings, we reached at the hypothesis that the antimicrobial peptide JH8194 we produced could have physiological functions on osteoblast cells, besides the inhibitory ability against *P. gingivalis*. The synthesized peptides immobilized on the titanium possessing the functions of both shortening the period for osseointegration and killing the bacteria would help to raise the ratio of success for dental implants.

Therefore, the purposes of this study were: i) to confirm the anti-bactericidal function of the titanium surface on which JH8194 was immobilized, according to the immobilization method described previously [8], and ii) to investigate the effects of Ti surface on which JH8194 was immobilized on the proliferation and differentiation of an osteoblastic cell line, MC3T3-E1.

## 2. Results and Discussion

### 2.1. *P. gingivalis* Biofilm Formation on the Titanium Surface Immobilized with JH8194

**Figure 1.**
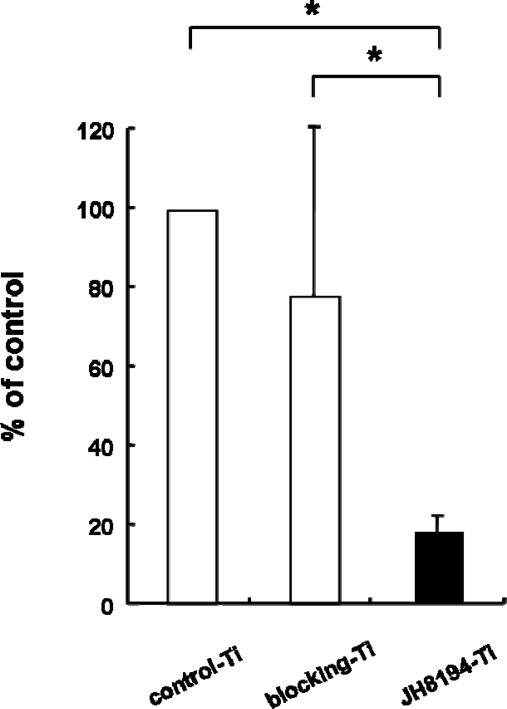
The effects of JH8194-Ti on *Porphyromonas* gingivalis were examined by ATP assay. *P. gingivalis* was grown on control-Ti, blocking-Ti or JH8194-Ti for four days. The assays were carried out on two independent occasions. Data represent the means ± SD of triplicate experiments. * p < 0.05.

Eighty percent of *P. gingivalis* viability was lost when *P. gingivalis* was grown on JH8194-Ti surface for four days, as compared with that grown on control-Ti. This inhibition was significant (ANOVA, p < 0.05). On the other hand, blocking-Ti, which contained no JH8194, did not affect on the colonization of *P. gingivalis* over the same period (Figure 1).

### 2.2. Effects of Soluble JH8194 on the Proliferation of MC3T3-E1 Cells

**Figure 2.**
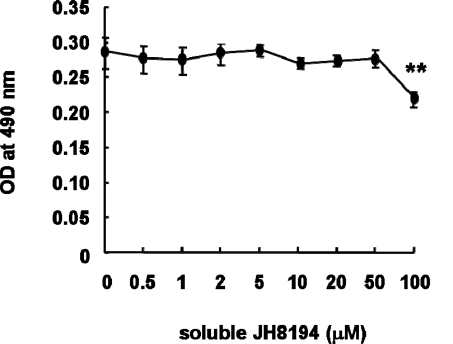
The effects of soluble JH8194 on the proliferation of MC3T3-E1 cells were investigated by MTS assay. The cells were cultured in the presence or absence of soluble JH8194 for four days. Soluble JH8194 was added to each well just once until the following assay. The MTS assay was done to examine the effects of soluble JH8194 on MC3T3-E1 proliferation. Independent experiments were repeated three times. Data represent the means +/– S.D of quadruplicate experiments.

The result of MTS assay showed that soluble JH8194 at the concentration range between 0.5 and 50 μM had no effect on the proliferation of the MC3T3-E1 cell, when the cells were exposed to soluble JH8194 for four days until the assay (ANOVA, p > 0.05) (Figure 2), whereas soluble JH8194 (100 μM) significantly inhibited the proliferation of the MC3T3-E1 cells, as compared with that of soluble JH8194-free control cell (ANOVA, p < 0.01) (Figure 2).

### 2.3. Effects of the Soluble JH8194 on the Levels of mRNAs for Runx2 and OPN in MC3T3-E1 Cells

**Figure 3.**
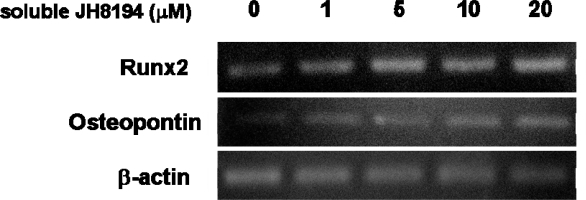
The effects of soluble JH8194 on the osteoblastic gene expressions were analyzed by RT-PCR. In the culture system, the cells were inoculated (day-0) and then reached at confluence in three days (day-3). By the two different types of duration of soluble JH8194, the alteration of the osteoblast-differentiation markers in MC3T3-E1 loaded with soluble JH8194 was analyzed. Concretely, the first pattern of soluble JH8194-duration was that soluble JH8194 was added to the cells on day-0 just after inoculation of the cells, followed by that the cells were successively cultured for seven days. The other one was that the exposure of soluble JH8194 to the cells, for the first time, started, when the cells became confluent (day-3). The cells were cultured, totally during seven days including the period until confluence. The addition of soluble JH8194 was done once until RNA isolation in both patterns in JH8194-duration. Total RNA was extracted from each cell to analyze the expression levels of Runx2 and OPN mRNA. Data are representative of three experiments.

To examine the effects of soluble JH8194 on the osteoblast differentiation, osteoblast-specific transcriptional gene markers; Runx2 and OPN in MC3T3-E1 cells exposed to soluble JH8194 were analyzed by RT-PCR. The results showed that soluble JH8194 enhanced the levels of mRNAs for Runx2 and OPN in MC3T3-E1, in a dose-dependent fashion, when the cells, for the first time, started to be impacted to JH8194 just after confluence (Figure 3). Soluble JH8194 at concentrations of 1, 5, 10 and 20 μM had no effect on the level of mRNA for β-actin, which was amplified as an internal control (Figure 3). On the other hand, in case of the addition of soluble JH8194 to the cells on day-0, no bands on the agarose gels corresponding to the amplified products of Runx2 and OPN except β-actin in MC3T3-E1 was detected by the RT-PCR method, under the same conditions of RT-PCR as the result of Figure 3 (data not shown).

### 2.4. Effects of the Immobilized-JH8194 on the Titanium Surface on the Levels of mRNAs for Runx2 and OPN in MC3T3-E1 Cells

**Figure 4.**
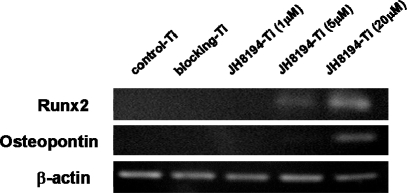
The effects of JH8194-Ti on the osteoblastic differentiation markers were analyzed by RT-PCR. The RNA was extracted from the cells cultured on control-Ti, blocking-Ti and JH8194-Ti for seven days after the cells were seeded. No supplement of soluble JH8194 was added to each well until RNA isolation. Data are representative of three experiments.

Next, to investigate the effects of JH8194-Ti on the osteoblast differentiation, MC3T3-E1 cells were grown for seven days on JH8194-Ti, JH8194-free blocking-Ti or control-Ti, which were prepared by the method as described in the Materials and Methods. The RT-PCR results revealed that the levels of mRNAs for Runx2 and OPN in MC3T3-E1 cultured on 20 μM JH8194-Ti were increased, as compared with those in the cells cultured on control-Ti, blocking-Ti and 1, 5 and 20 μM JH8194-Ti (Figure 4). JH8194-Ti of 5 μM slightly enhanced the mRNA expression of Runx2 (Figure 4). All titanium had no remarkable effect on the mRNA expressions of β-actin (Figure 4).

### 2.5. Effects of the Immobilized-JH8194 on the Titanium Surface on the Activity of ALPase in MC3T3-E1 Cells

**Figure 5.**
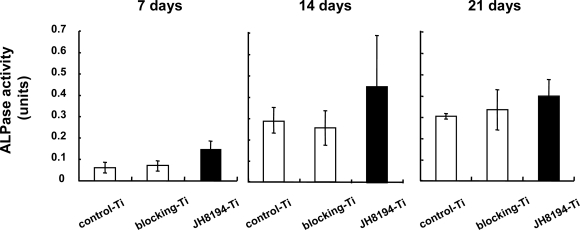
The effects of JH8194-Ti on ALPase activity. ALPase activity and total protein in the solved supernatant with 0.2% TritonX-100 in saline of each cell grown on control-Ti, blocking-Ti and JH8194-Ti during 7, 14 and 21 days were measured in accordance with the method described in Materials and Methods. ALPase activity per one mg protein (units) was calculated. Data represent the means ± SD of triplicate experiments. Three independent experiments were carried out. (ANOVA, p >0.05)

Finally, ALPase activity was analyzed. The ALPase activity per the protein in MC3T3-E1 cells cultured on 20 μM JH8194-Ti for 7, 14 and 21 days was greater than those in the cells on control-Ti and blocking-Ti (Figure 5), but, there was no significance between the samples (ANOVA, p > 0.05).

### 2.6. Discussion

Histatin 5 is the most potent member of the family and renders most pathogenic *Candida* species non-viable *in vitro* at physiological concentration [13]. It was reported that histatin 5 interacted with bacterial cells of *P. gingivalis* [12], which is often detected in the tissue and titanium surface around failed dental implants [4]. Collectively, these findings suggested that JH8194, which was derived from histatin, similarly would bind to the *P. gingivalis* cell wall and kill it, as well as killing *Candida albicans* [9]. In the present study, the result of biofilm assay using *P. gingivalis* proved that JH8194 immobilized on the titanium surface by our method inhibited the formation of *P. gingivalis* biofilm, as we predicted (Figure 1). Bone morphogenic proteins (BMPs) are generally known to increase bone formation, and BMPs released from atelopeptide type I collagen (carrier) stimulated a bone response in peri-implants [14]. However, BMP immobilized on titanium did not increase peri-implant bone formation [15]. These reports suggest that the conformation and/or activity of the proteins after the immobilization process to titanium surface are very important for the function of the immobilized protein. In the case of immobilization of synthetic peptides on a titanium surface, the function of the peptides may be attributed to stability of the conformation and/or activity of the synthetic peptides. It is generally accepted that numerous antimicrobial peptides have an alpha-helical structure, and the majority are cationic and amphipathic [9]. Our previous report indicated that candicidal activity of JH8194 was due to its alpha-helical propensity [9]. The inhibitory action of immobilized-JH8194 on the titanium surface against *P. gingivalis* may similarly involve the alpha-helical structure of JH8194, which remained on the titanium surface after the immobilization of the present study.

To verify the hypothesis of whether a newly antimicrobial peptide we produced, JH8194, could have some effects on the osteoblast cells or not, MC3T3-E1 cells were cultured in the presence or absence of soluble JH8194. This cell line is a normal mouse osteoblast-like cell, which is not derived from tumors like the osteogenic sarcoma. It expresses ALPase and forms calcification under usual culture conditions [16]. Therefore, this was useful for the present *in vitro* experiments.

The results of the MTS assay in Figure 2 suggested that the high concentration of soluble JH8194 had a cytotoxic effect on MC3T3-E1 cells. This was consistent with the finding of the cytotoxicity of the peptide derived from histatin and lactoferricin on MC3T3 [11]. Murakami *et al*. reported the synergistic increase of the chondrocyte-proliferation by histatin 5, under the stimulation of epidermal growth factor [10]. Although the proliferation of MC3T3-E1 was not affected by soluble JH8194 stimulation at the low concentrations (Figure 2), there still remained the possibility that JH8194 could increase the osteoblast-proliferation in cooperation with other cytokines, since JH8194 was derived from histatin. Taking into consideration the findings of the proliferation assay, we used four concentrations of soluble JH8194, *i.e.*, 1, 5, 10 and 20 μM, in order to examine the molecular responses of MC3T3-E1 against soluble JH8194.

As the results of Figure 3 show, soluble JH8194 surprisingly enhanced mRNA expressions of initial osteoclast differentiation markers; Runx2 [17] and OPN [16] expressed by MC3T3-E1 cells, in a dose-dependent manner. However, no similar enhancement of the genes was observed, when the exposure of soluble JH8194 started at the inoculation (data not shown). The function of soluble JH8194 on the osteoblast differentiation may be dependent on the conditions of the cells, although the mechanisms of the action by soluble JH8194 on the osteoblast cells were unclear. The extracellular matrix and its derivative including collagen [18,19], fibronectin [20], vitronectin [20] and RGD peptide [21] enhanced the cell attachment on and spreading to the titanium surface, which could consequently lead to the cell proliferation and differentiation. Different from the extracellular matrix, soluble JH8194 may bind to the cell surface and directly induce osteoblast differentiation through the signal transduction, which might be likely to the function of growth factors such as BMP [14].

We previously reported that OPG immobilized on the titanium surface by the same immobilization-method used in the present study was slowly released from the titanium surface [8], which would support a possibility that immobilized-JH8194 was a slow carrier for JH8194. Therefore, we attempted to examine the effect of immobilized-JH8194 on the osteoblast differentiation. To our surprise, 20 μM JH8194-Ti surface accelerated the initial differentiation (Figure 4), as soluble JH8194 did. Similarly, there were increases of ALPase activity, which is a maturation-stage marker during osteoblastogenesis [22], by immobilized-JH8194, but those were not significant (Figure 5). At least, the data in the present study support to prove the hypothesis that JH8194-immobilizad on titanium specimens could enhance the initial differentiation of osteoblast cells, consequently resulting in slight increase of ALPase activity. It was speculated that the functional JH8194 might remain on the titanium surface without degradation, or that immobilized-JH8194 could be slowly released from the surface of titanium. Thus, JH8194-titanium produced by the present method may be used as a carrier for a slow delivery of JH8194, which was a new factor for inducing the initial osteoblast differentiation. Further research may include studies in animals to validate the findings. On the other hand, it was difficult to examine whether mechanical forces simulating insertion of dental implants cause abrasion of JH8194 binding to titanium surface or not, since the proper *in vitro* methodology was not still established. However, the surgical procedure was reported in order to avoid abrasion of the material coating on the titanium surface during insertion [15]. In addition, JH8194 was immobilized through covalently bonding to the titanium surface, hence the risk of drop out of the peptides by abrasion seems to be minimal. Further study, such as an *in vivo* assay should clarify the issue.

Taken together, the results in the present study suggest that JH8194, an antimicrobial peptide derived from histatin we produced, can be immobilized on a titanium surface by our immobilization method and that it retained its antimicrobial activity after the immobilization process. Moreover, it was suggested that JH8194-immobilized on titanium specimens initially enhanced not proliferation but differentiation of the osteoblast cells. Although further studies are required to understand the two mechanisms of JH8194 in inhibiting *P. gingivalis* biofilm formation and inducing the osteoblast differentiation, JH8194 is a candidate for surface substrates in dental implants in order to enhance the acquisition of osseointegration and decrease infection, leading to an increased ratio of treatment success. In the next step, *in vivo* experiments using dog mandibles and titanium screw fixtures which surfaces are entirely or partly immobilized with JH8194 will help to prove that JH8194 remaining on the surface of the fixture inserted according to the surgical procedure for avoiding friction [15] can protect infection and simultaneously accelerate bone formation around dental implants.

## 3. Experimental Section

### 3.1. Purity of a Synthetic Peptide; JH8194

The peptide JH8194 was synthesized at Greiner Bio-One Co., Ltd. (Tokyo, Japan) [9].

### 3.2. Immobilization of the Synthetic Cationic Peptide; JH8194, on Titanium Surfaces

Pure wrought titanium (cp-titan) disks (JIS, Japan Industrial Specification H 4600, 99.9 mass% titanium, diameter 15 mm; Kobelco, Kobe, Japan) were purchased and used in the experiments. Preparation and immobilization of JH8194 on the titanium surface was carried out in accordance with the previous studies [8,23–26]. In brief, titanium specimens were immersed in 5% γ-aminopropyltriethoxysilane in acetone for 15 min at room temperature and washed with acetone. Subsequently, specimens were treated with 5% glyoxylic acid monohydrate for 2 hours, and then washed with ultra-pure water. Then the surfaces of the specimens were treated with 0.4% sodium borohydride (NaBH_4_) for 24 hours, to reduce the imine to amine groups. After this series of pre-treatment, the titanium was washed with ultra-pure water and autoclaved. Then, the carboxyl groups on the surfaces of specimens were activated with *N*-hydroxysuccinimide (NHS)/*N*-ethyl-*N’*-(3-dimethylaminopropyl)-carbodiimide (EDC) (BiaCore AB, Uppsala, Sweden), and treated with 1, 5, 10 and 20 μM JH8194 in sodium bicarbonate buffer (pH 8.0) for 30 min at 37 °C, to immobilize JH8194 on the surface. After washing with phosphate-buffered saline (PBS) to remove any excess JH8194, the activated carboxyl groups were blocked by a 5-min treatment with 1 M ethanolamine-HCl (BiaCore AB) (JH8194-Ti). Blocking-Ti was prepared by treatment of titanium specimens with 1 M ethanolamine-HCl immediately after the carboxyl groups were activated by NHS/EDC treatment (blocking-Ti). Untreated-titanium specimens were also used as control specimens (control-Ti). The diameter of the cylindrical-shaped titanium disc fit within the well of a standard 24-well tissue culture plate [27].

### 3.3. Microorganism and Growth Conditions

*P. gingivalis* isolated from the oral cavity of a patient was used in this assay. A loopful of the microbial was inoculated in Brain Heart Infusion broth (BHI, Difco, Detroit, USA) containing 5.0 μg/mL hemin and 1.0 μg/mL menadione, and grown anaerobically at 37 °C. After seven days culture, the microbial was harvested in the late exponential growth phase, washed twice with PBS and resuspended to a final concentration of 10^8^ cfu/mL by a spectrophotometeric method.

### 3.4. Biofilm Assay

The colonization assay was conducted as follows. After preparation for Ti plates (control-Ti, blocking-Ti and JH8194-Ti), 50 μL of microbial suspension (1 × 10^8^ cfu/mL) was inoculated into each titanium specimen to promote microbial adherence and colonization at 37 °C for two hours. Subsequently, 2.0 mL of BHI supplemented with hemin and menadione was carefully dispensed into each well, and incubated for four days at 37 °C in an anaerobic condition. Afterwards each specimen was washed carefully by rising three times with PBS to remove loosely adherent organisms, then 1.0 mL of PBS was added and biofilms diffused by pipetting. The resultant biofilm suspension was then inoculated into a cuvette and subjected to optical density (OD)-measurements to quantify the microbial growth in each well.

### 3.5. Culture of MC3T3-E1

The MC3T3-E1 cell line was purchased from the European Collection of Cell Cultures (ECACC, Wiltshire, UK). MC3T3-E1 cells were cultured in α-MEM supplemented with an antibiotic mixture (Invitrogen, Carlsbad, CA, USA), 10% fetal bovine serum (FBS) (Biological Industries, Haemek, Israel) and 50 μg/mL l-ascorbic acid (Sigma, St. Louis, MO, USA). MC3T3-E1 cells were maintained for each experiment at 37 °C under 5% CO_2_/95% humidified air. During culture, the medium was refreshed at three-day interval.

### 3.6. MTS Assay

MC3T3-E1 cells were seeded onto 96-well plate (Becton Dickinson, Franklin Lakes, NJ, USA) at a density of 1.0 × 10^4^ cells/well and cultured for four days in the presence of soluble JH8194 at the range of 0 to 100 μM. Suspended cells were removed by gentle rinsing with PBS and the number of adherent cells remaining in each well was then quantified using a coupled enzymatic assay, which resulted in the conversion of a tetrazolium salt into a red formazan product (MTS assay, CellTiter 96 Aqueous Non-Radioactive Cell Proliferation Assay, Promega, Madison, WI, USA) [28]. Recording of the absorbance at 490 nm in the MTS assay was carried out using a microplate reader (Bio-Rad, Hercules, CA, USA).

### 3.7. Reverse Transcriptase-Polymerase Chain Reaction (RT-PCR) Analysis

MC3T3-E1 cells were seeded onto 24-well plates (Becton Dickinson) or the surface of titanium specimens placed on the bottom of the same plates, at a density of 5.0 × 10^4^ cells/well or titanium specimen, and maintained for seven days. Total RNA was extracted using TRIzol reagent (Invitrogen), and first-strand cDNA was synthesized from total RNA (100 ng) using ReverTra Ace reverse transcriptase (Toyobo). The cDNA was then amplified by BIOTAQ DNA polymerase (Bioline, Randolph, MA, USA). For each gene, a cycle curve experiment was performed, and the optimal number of PCR cycles was selected according to the results. Osteoblastic gene expressions for Runx2 and OPN were analyzed by RT-PCR. The gene encoding β-actin was used as internal control. The sequences of forward and reverse primers for OPN were 5′-ACACTTTCACTCCAATCGTC-3′ and 5′-TGCCCTTTCCGTTGTTGTCC-3′. The sequences of other primers used in these analyses were previously described [17].

### 3.8. Measurement of Alkaline Phosphatase Activity

To determine the effect of titanium immobilized with JH8194 on alkaline phosphatase (ALPase) activity was analyzed using ALPase activity kit (p-Nitrophenyl Phosphate Liquid Substrate System, Sigma). Cells were seeded and the medium was replaced at three-day intervals until analysis. ALPase activity was measured in each cell layer after 7, 14 and 21 days in culture. Cell layers were gently rinsed three times with PBS. Each cell layer supplemented with 0.2% TritonX-100 in saline was homogenized three times (10 seconds/time) on ice, using the micro-homogenizer (ULTRA-TURRAX^®^, As One, Osaka, Japan), and the homogenates were then centrifuged for 5 min at 13,000 × g in order to eliminate the dissolved fractions such as the cell debris, prior to collection of the supernatant containing the alkaline phosphatase. The isolated samples of supernatants and the standard enzyme of calf alkaline phosphatase were incubated with the substrate for 30 min at 37 °C, in accordance with the indicated procedures. The optical density was measured at 405 nm in the microplate reader (Model 550, Bio-Rad Laboratories, Tokyo).

Estimation of protein content was carried out using a Coomassie Plus Assay Kit (Thermo Fisher SCIENTIFIC, Yokohama). The optical density was measured at 595 nm in the microplate reader (Model 680, Bio-Rad), according to the procedure. Collectively, the enzyme activity was expressed as units per milligram of total proteins, based on the data by ALPase activity and Coomassie Plus Assay kits.

### 3.9. Data Analysis

Differences among average values of groups were subjected to a one-way analysis-of-variance (ANOVA) and Tukey’s multiple range test.

## 4. Conclusions

This study suggested that a new antimicrobial peptide JH8194-immobilized titanium may be a new strategy to protect early infection in replacement surgery of implant fixtures and to shorten the period of acquisition of osseointegration.
